# Robust estimation of hemo-dynamic parameters in traditional DCE-MRI models

**DOI:** 10.1371/journal.pone.0209891

**Published:** 2019-01-03

**Authors:** Mikkel B. Hansen, Anna Tietze, Søren Haack, Jesper Kallehauge, Irene K. Mikkelsen, Leif Østergaard, Kim Mouridsen

**Affiliations:** 1 Center of Functionally Integrative Neuroscience and MIND*Lab*, Institute of Clinical Medicine, Aarhus University, Aarhus, Denmark; 2 Inst. of Neuroradiology, Charité University Medicine Berlin, Berlin, Germany; 3 Department of Clinical Engineering, Aarhus University Hospital, Aarhus, Denmark; 4 Department of Oncology, Aarhus University Hospital, Aarhus, Denmark; 5 Department of Medical Physics, Aarhus University Hospital, Aarhus, Denmark; Ludwig-Maximilians University (LMU), GERMANY

## Abstract

**Purpose:**

In dynamic contrast enhanced (DCE) MRI, separation of signal contributions from perfusion and leakage requires robust estimation of parameters in a pharmacokinetic model. We present and quantify the performance of a method to compute tissue hemodynamic parameters from DCE data using established pharmacokinetic models.

**Methods:**

We propose a Bayesian scheme to obtain perfusion metrics from DCE MRI data. Initial performance is assessed through digital phantoms of the extended Tofts model (ETM) and the two-compartment exchange model (2CXM), comparing the Bayesian scheme to the standard Levenberg-Marquardt (LM) algorithm. Digital phantoms are also invoked to identify limitations in the pharmacokinetic models related to measurement conditions. Using computed maps of the extra vascular volume (v_e_) from 19 glioma patients, we analyze differences in the number of un-physiological high-intensity v_e_ values for both ETM and 2CXM, using a one-tailed paired t-test assuming un-equal variance.

**Results:**

The Bayesian parameter estimation scheme demonstrated superior performance over the LM technique in the digital phantom simulations. In addition, we identified limitations in parameter reliability in relation to scan duration for the 2CXM. DCE data for glioma and cervical cancer patients was analyzed with both algorithms and demonstrated improvement in image readability for the Bayesian method. The Bayesian method demonstrated significantly fewer non-physiological high-intensity v_e_ values for the ETM (p<0.0001) and the 2CXM (p<0.0001).

**Conclusion:**

We have demonstrated substantial improvement of the perceptive quality of pharmacokinetic parameters from advanced compartment models using the Bayesian parameter estimation scheme as compared to the LM technique.

## Introduction

The non-invasive visualization of tissue perfusion represents a valuable tool in primary diagnosis and follow-up of several diseases. Imaging biomarkers that directly or indirectly address perfusion can be obtained by for example computed tomography (CT), magnetic resonance imaging (MRI), or positron emission tomography (PET). Of these fundamentally different measurement techniques, MRI has received particular attention since ionizing radiation is avoided and the soft tissue contrast is excellent. Perfusion-weighted MRI is used to characterize various pathologies, as for example ischemic stroke[1] or cancer diseases in both the brain and other parts of the body[2].

T2-based MRI perfusion, termed dynamic susceptibility contrast (DSC), is widely implemented in neuroimaging and has proven useful in stroke and brain tumor diagnostics. This method is, however, hampered by its limited applicability when tracer leaks out of the vasculature, as in non-brain tissue or when the blood-brain-barrier (BBB) is disrupted. In this case, non-quantifiable T1-effects confound the conversion of signal data to tracer concentrations. In addition, DSC MRI is not a quantitative method due to the different T2 relaxivities of blood and tissue and the non-linear relationship between signal change and concentration in blood. Hemodynamic parameters, such as blood flow and blood volume, are therefore only relative. The T1-based methodology is termed dynamic contrast enhanced (DCE) and is less affected by these limitations. First, the short echo times of about 1ms result in negligible T2-effects and allow straightforward generation of tracer concentration curves from observed signal changes. Hence, DCE MRI may be used in any tissue, and has been the preferred tool to quantify BBB integrity in cerebral diseases. Second, the similar T1-relaxivities in arterial blood and tissue and linear relationship between T1 relaxation and tracer concentration[3] better allow absolute parameter quantification. DCE MRI is, however, challenged by the low blood content in normal brain tissue resulting in minimal signal changes, which, in conjunction with stochastic experimental noise, makes parameter estimation difficult. In addition, while it is potentially possible to obtain quantitative measurements of blood flow and blood volume from DCE measurements, these quantities must be inferred from measured data by assuming a model of the tissue and vascular system underlying each particular voxel. Typically, these models are interpreted as so-called compartmental systems [4–7], which, depending on their complexity, may yield information on overall flow, inter-compartmental flow, blood volume, accessible extra-cellular volume fractions, etc. Indeed, models range from simple one-compartment models such as the Patlak model [8] or the Tofts model [9] over two-compartment models [4, 7] to generic multi-compartment models [10]. The selection of model has received much attention [11, 12], but is only mentioned here for completeness, while referring to other works for details [12, 13].

Furthermore, parameter reproducibility may be challenging due to complex experimental settings, the conversion of the signal changes to concentration changes, and determining the functional form of the feeding vessel, the so-called arterial input function (AIF), see for example [14–16] and references therein. These reproducibility challenges are very important in the setting of multicenter trials, where for example early treatment changes of anti-cancer drugs have to be assessed and where accurate quantitative measurements are crucial. This may, however, be less critical in routine clinical use, where the qualitative structure of the parameter maps is usually in focus. The aim of this paper is to provide a characterization of the clinical usability of two widely known models; the extended Tofts model (ETM) [5] and the two-compartment exchange model (2CXM) [7].

One central challenge in the model-based analysis of perfusion data is the fitting of the model parameters to the observed data. Due to factors such as image noise, low signal, and large parameter space compared to the number of observations, such fitting might be difficult, in particular when standard methodologies such as the well-known Levenberg-Marquardt (LM) approach are used. Therefore, we investigate a Bayesian method (BM), with posterior parameter and noise distributions determined through a variational Bayesian algorithm, which has previously been shown to increase the reliability of DSC parameter estimation [17]. We assess the performance of BM and LM algorithms in the ETM and 2CXM models that are widely used in DCE MRI. Initially, we assess the performance of the proposed algorithm for the ETM and 2CXM using digital phantom data, for which the ground truth is known, while also providing a first assessment of possible bounds on the reliability of parameter estimation. In the second part, we illustrate the differences of DCE parameter maps, based on LM and BM, respectively, by providing clinical examples of patients with brain tumors and cervical cancer.

## Materials and methods

### Theory

As the primary focus of this work is the assessment of parameter estimation, we only provide a short overview on DCE MRI and refer otherwise to [6, 7] for elaborate reviews. In DCE MRI, gadolinium-based contrast agents are injected intravenously, and the resulting measured signal changes are interpreted as T1 relaxation effects. Assuming a linear relationship between the tracer concentration and the T1 relaxation, the signal intensity curves for each voxel may be translated into concentration-time curves (CTC), see Appendix A in S1 File.

The CTCs resulting for each of the tissue voxels are targets for further analysis. Typically, the analysis of the CTCs takes onset in a compartmental model, where the vasculature represents one compartment and the extra-vascular extra-cellular space (EES) another. Common to models described herein is that the CTC is represented by a convolution integral, where a known arterial input function (AIF) is convolved with an unknown impulse response function, as

$$C_{t}(t)=F_{p}\int_{0}^{t}C_{p}(\tau )R(t-\tau |\theta )d\tau$$ (1)

Here, we have adopted the notation from [7], defining C_t_(t) as a measured tissue CTC, C_p_(t) a known plasma CTC, typically from an artery, F_p_ the intravascular flow, R the residue function, and θ a set of model parameters. Traditionally, analysis of DCE MRI data proceeds through the construction of a model for the indicator passage described by the residue function. In this work, we will consider two extensively used models, (i) the ETM(5) and (ii) the 2CXM(7), which may be written as

$$\mathrm{ETM}:\;R(t)=\exp\left(-\frac{K_{trans}t}{v_{e}}\right)+\frac{v_{p}}{K_{trans}}\delta (t)=\exp\left(-k_{ep}t\right)+\frac{v_{p}}{K_{trans}}\delta (t)$$ (2)

$$2\mathrm{CXM}:\;R(t)=\exp\left(-tK_{+}\right)+E_{-}(\exp\left(-tK_{+}\right)-\exp\left(-tK_{-}\right))$$ (3)

In the ETM, K^trans^, v_e_, v_p_ are model parameters, while δ denotes the Dirac delta function. K^trans^, in turn represents intra- to extravascular tracer exchange and bulk flow, while v_e_ and v_p_ represent extravascular and plasma volume fractions, respectively, In the present implementations, k_ep_ is determined rather than v_e_ due to, in our hands, better independent parameter estimation. In the 2CXM, F_p_, K_±_, and E_-_ are model parameters, from which compartmental flows and volumes may be obtained, including v_p_, v_e_, and an inter-compartment transfer constant denoted K_1_ in the following. Note that K_±_ and E_-_ can be equally well represented in terms of mean transit-times T_E_, T_P_, and T_B_ for the extra-vascular, plasma, and combined compartments, respectively [7]. The transformation between the two representations is straightforward, but in our hands, the rate-constant expression was found to be more stable, hence making this the natural choice. For future reference, we denote the mean transit-times in the individual compartments T_E_, T_P_, and T_B_ for the extra-vascular, plasma, and combined compartments, respectively in accordance with established convention [7]. We note that recent studies on model selection have favored the compartmental tissue uptake model (CTUM) for cervical cancer patients [11, 18]. The CTUM is, however, a sub-model of the 2CXM and, since the present work is focused on model fitting rather than model selection, we restrict the following analyses to the well-known 2CXM and ETM models.

The fundamental problem addressed in this work is the estimation of parameters in a model, which may generally be written as

y(t)∼M(t|θ)+ϵ (4)

where y is observed data, M denotes a model fitted to these data through parameters θ, and ε is an error term, representing stochastic experimental error. The model is in this case given by a convolution integral as those represented in Eqs (1)–(3), i.e. we elected to fit the model to the CTC rather than including the conversion from signal to concentration in the model as well.

The common approach to solving deconvolution problems with residue functions with closed mathematical expressions is to apply iterative minimization of the residual sum-of-squares between the observed and estimated curves. Whereas LM is a common choice for such problems, the non-linear character of the fitting problems in DCE MRI is, however, often difficult to handle for standard LM algorithms, resulting in un-physiological parameter estimates. This has led to alternative formulations, of which a recently developed Bayesian approach has shown promising results in DSC MRI [17, 19].

BM attempts to estimate model parameters by assuming that they follow a multivariate normal distribution. Hence, the parameters are described through a multivariate mean and covariance. The problem to solve can be completely stated by assuming that the unobserved error in Eq (4) is Gaussian with zero mean and covariance C_ε_. The overview of the algorithm is as follows: First, the parameter distribution is initialized through a prior mean and covariance, which is subsequently used in an adaptive scheme to maximize a log-likelihood function through an expectation-maximization (EM) algorithm. The maximization step adaptively determines C_ε_. While the details of the algorithmic framework may be found in [17], the practical details of the present implementation must be stated. To that end, we used a prior covariance matrix with diagonal elements (0.1, 10, 0.1, 10) for the parameters (F_p_, F_p_/v_e_, v_p_, delay) for the ETM, while the prior covariance matrix (0.1, 1, 10, 1, 10) for the parameters (F_p_, T_P_, T_E_, T_B_, delay) was used for the 2CXM. The prior means were obtained individually for each voxel, under the assumption that T_P_ was 5 seconds, T_B_ was 10 seconds, T_E_ was 100 seconds, and the delay was 1 second. F_p_ was calculated as the blood volume (CBV) divided by T_P_ (CBV/T_P_), where CBV in turn was obtained as the area under the concentration curve. In addition, the algorithm is run for a maximum of 32 iterations, which has been found to be sufficient in applications. Convergence is checked after a minimum of 15 iterations via the length of the update step (threshold: step length less than 1.0*10^−4^).

In this work, we consider implementation of the Bayesian parameter estimation algorithm as applied to ETM and 2CXM and compare it to the performance of the well-known LM algorithm.

We note that other research groups have previously published work on Bayesian methodology in the context of DCE hemodynamic parameter estimation [13, 20, 21], which are similar in spirit to the one presented in this work. While similar in spirit, the algorithm presented here utilizes patient specific measured AIFs rather than, which is quite common in DCE studies, a bi-exponential fit to a measured input function or a universal input function [22]. In addition, delay between site of measurement of the AIF and a particular tissue curve is an adaptive parameter in our model alongside the actual hemodynamic parameters, such as F_p_ and v_e_, which has been included in some works [13, 23, 24], whereas it seems not to be for others [20, 21].

### Simulations

We assess performance of the two algorithms through simulated AIFs and tissue signal intensity curves, which in turn are obtained from CTCs modeled according to Eqs (1)–(3). The arterial CTC is constructed based on the functional form by Parker *et al* [22], while the tissue CTCs are obtained by convolution of the arterial CTC with residue functions from ETM or 2CXM. The one-dimensional convolution is performed on longitudinally up-sampled discrete data and the resulting tissue curve subsequently down-sampled to the desired temporal resolution. We assume a linear relation between the observed R_1_ change and the concentration [3], i.e.

$$R_{1}(c(t))=R_{1}+r_{1}c(t)$$ (5)

where c(t) is the tracer concentration as a function of time, R_1_ the tissue specific longitudinal relaxation rate, and r_1_ the relaxivity. Note that R1 is inversely proportional to the T1 relaxation time, i.e. T1 = 1/R1. Traditionally, DCE MRI has been used as a source for quantitative measurement of hemodynamic parameters, given the selection of a biophysical model. One central component in the analysis is, however, the pre-contrast T1 value of the voxel under consideration. In the simulation studies, we assume an arterial T1 value of 1.66s [25, 26], while tissue voxels are constructed based on a T1 value of, unless stated otherwise, 1 second, which is close to observed white matter values at 3T [27].

The arterial and tissue CTCs are converted to MRI intensities through a steady-state sequence, with parameters attaining experimentally achievable values (flip angle (FA) = 25^∘^, repetition time (TR) = 3ms, echo time (TE) = 1ms, r_1_ = 3.6 ms/mM, and time between dynamic acquisitions = 1.5s). Random noise is added according to

$$S^{wn}(t)=|S^{wn=0}(t)+\frac{S^{wn=0}(t=0)}{SNR}(X^{R}+X^{I})|,$$ (6)

where S^wn^(t) and S^wn = 0^(t) are the MR signals with and without noise, SNR is a signal-to-noise level, and X^R^ and X^I^ are random Gaussian distributed vectors with zero mean and unit standard deviation. The |·| operator denotes the norm of the complex number, resulting in a signal with Rician [28] noise.

### Digital phantom images

MRI signal phantoms are constructed with three spatial and one temporal dimension. This allows for three of the model parameters to be varied across the spatial dimensions, while the fourth dimension is reserved for the temporal evolution of the signal. The spatial size of the digital phantom is 128x128x8, where the first slice contains realizations of AIFs and the remaining slices reserved for tissue curves. Each of the tissue slices consists of 7x7 = 49 squares, which in turn consists of 14x14 = 196 voxels. The squares are separated by four voxels containing zeros, while a frame of three zero voxels is used. Within one square, the underlying CTCs are identical, while random Rician noise is added individually for each generated MRI signal intensity curve according to Eq (6). Since the ground truth is known for the phantoms, quantification of performance through systematic (bias)–and random (standard deviation, SD) deviance from the known ground truth is possible through the standard squared bias/variance decomposition of the mean squared error [17], i.e.

$${\mathrm{Variance}:\;\sigma }^{2}=\frac{1}{N_{voxel}}\sum_{i=1}^{N_{voxel}}{\left(y_{i}-\hat{y_{i}}\right)}^{2}$$

$$\mathrm{Bias}:\;bias=\frac{1}{N_{voxel}}\sum_{i=1}^{N_{voxel}}\left(y_{i}-\hat{y_{i}}\right)$$

Averaging the absolute bias or SD over all squares enables summarizing a hemo-dynamic parameter map obtained from a complete digital phantom by two numbers.

We construct phantoms for ETM and 2CXM, respectively, simulating signal-to-noise ratios (SNR) of 2, 5, 10, 20, 30, 40, 50, 60, 80, and 100. The temporal resolution was 1.5 seconds. For ETM, we vary K^trans^, k_ep_, and v_p_ independently across the three dimensions, whereas for 2CXM F_p_, v_p_, and v_e_ vary independently, while K1 is chosen as 10% of the F_p_ value. Table 1 presents the parameter ranges used in the simulations. The model used for generating each phantom was also used for the estimation of the parameters.

**Table 1:** Parameter ranges used in the simulations.

Model	K^trans^ or F_p_ ml/100g/min	K_ep_ or K_1_ ml/100g/min	V_e_ ml/100g	V_p_ ml/100g	Delay (s)
ETM	[12; 84]	[0.17; 8.4]	[10; 70]	[1; 7]	[0; 9]
2CXM	[12; 84]	[1.2; 8.4]	[10; 70]	[1; 7]	[0; 9]

### Patient data–cerebral brain cancer

We present sample results for 6 patients (4 males, 2 females), diagnosed with glioblastoma (GBM*)*, following the World Health Organization (WHO) classification scheme [29]. The patients underwent DCE MRI using a turbo fast low-angle shot (turbo-FLASH) technique [30] as part of the clinical protocol (TR = 3.51ms, TE = 1.7ms, flip angle = 25 degrees, in-plane field-of-view (FoV) = 220x220mm^2^, acquisition matrix = 128x128 voxels). A total of 15 slices were acquired at each time-point in a 75mm slab with the tumor in the center of the slab, yielding a voxel size of 1.72x1.72x5mm^3^. A total of 150 dynamic acquisitions were acquired at an interval of 2.03s, resulting in a total DCE acquisition time of 5:04 (min:sec). The injection scheme consisted of a single injection of 0.05mmol/kg gadobutrol at 2.5ml/s, flushed with 30ml saline at the same rate. All patients were scanned on a Philips Achiva 3T MRI scanner (Philips, Best, Netherlands). The patient data were collected as part of a clinical study, approved by the Danish Committee on Health Research Ethics (local committee: Central Denmark Region), and written informed consent was obtained from all subjects.

In addition, we compare the appearance of the computed v_e_ maps through the number of un-physiological high-values, comparing the LM and BM methods for both ETM and 2CXM using a one-tailed paired t-test assuming unequal variance in the samples.

### Patient data–cervical cancer

We present sample results for 5 patients diagnosed with advanced cervical cancer (stage 3 IIB/2 IIIB). DCE imaging was performed as part of the MRI protocol imaged at a 3T Philips Achieva (Philips, Best, Netherlands). DCE was acquired using a centric sequence [18] (TR/TE/T_sat_ = 2.9/1.4/25ms, flip angle = 10 degrees, in-plane FoV = 400x400 mm^2^, image matrix = 176x176 voxels, 12 slices, voxel size = 2.27x2.27x6mm^3^, inter-slice gap = 3mm, total coverage: 400x400x105mm^3^). A total of 120 dynamic acquisitions were collected at an interval of 2.1s resulting in a total acquisition time of 4:12. After an initial 18 dynamic acquisitions used to determine baseline signal, 0.1 mmol/kg Gd-based contrast agent (Dotarem, Guerbet) was injected using a power injector at a rate of 4 ml/s followed by 50 ml of saline injected at the same rate.

### Data processing

All data in this study, both simulated and patient data, have been processing by the same pipeline, which is briefly presented here. After loading the data from DICOM images, motion correction was performed using a 12-parameter affine transformation, correcting all temporal volumes to the first temporal volume. The motion correction step was performed using the Statistical Parametric Modeling toolbox version 12 (SPM12). Next, the pre-contrast baseline was determined automatically, by fitting a time-shifted gamma-variate to the mean intensity curve and selecting the bolus initiation as two timepoints before the point of maximum change. This fitting used a LM procedure. With the pre-bolus determined, concentration-time curves were calculated from the intensity-time curves as outlined in Appendix A in S1 File. A fixed prebolus T1 value of 1 second was, except stated otherwise, used for all but the AIF, where a prebolus T1 value of 1.66 seconds was used. An experienced neuroradiologist (AT) performed manual selection of the AIF using in-house developed software (pgui, http://cfin.au.dk/software/pgui/). Calculations of parameter maps were performed using in-house developed software (pgui, http://cfin.au.dk/software/pgui/). The LM algorithm used was the one supplied by the curve fitting toolbox available in MATLAB (Mathworks, Natick), while the BM was an in-house implementation. All steps of the described pipeline were implemented in MATLAB.

## Results

### Simulations

**Fig 1:**
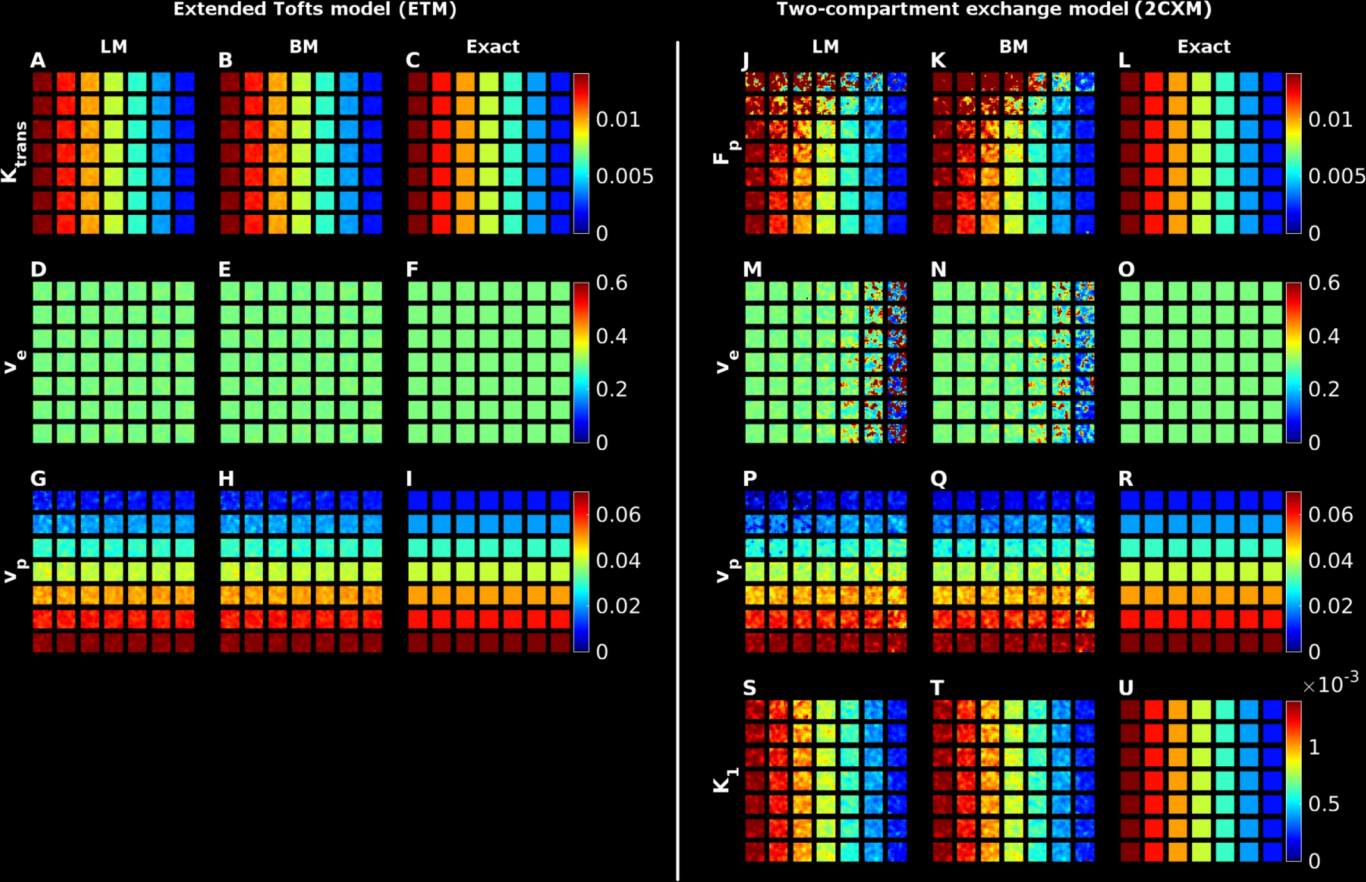
Illustrations of parameter plots using the Bayes or LM method for the extended Tofts model (panels A to I) and the two-compartment exchange correlation model (panels J to U). Each row in the two model panels represents a given parameter. The first two columns for each row represent the BM and LM parameter estimates, while the third column present the ground truth parameter map (denoted ‘Exact’ in the figure).

**Fig 2:**
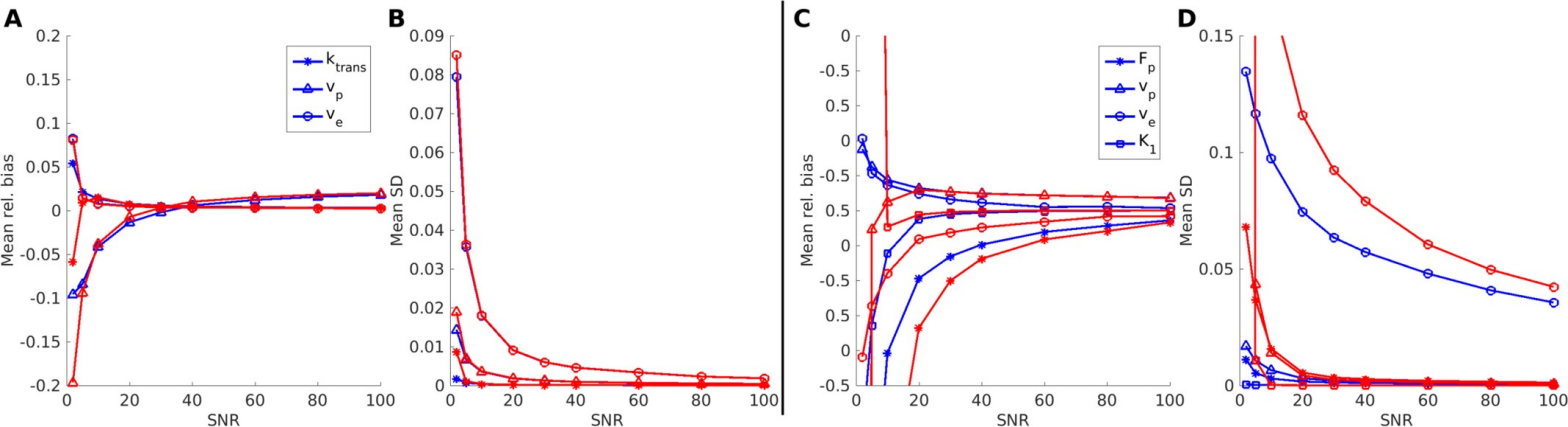
Bias and standard deviation plots of entire digital phantoms for the Tofts and 2XC model parameters as a function of SNR. In panels A and B, the bias and standard deviation for the ETM model parameters are displayed as a function of SNR, while the similar plots for the 2XC model parameters are presented in panels C and D. The Bayes and LM parameters are displayed in blue and red, respectively and the insert in A and C define the symbols used for the model parameters.

In Fig 1, we present examples of parameter maps obtained from digital phantom data. The temporal evolution of the signal was simulated for 5 minutes, which is typical of standard 3D DCE measurements in for example cancer protocols. For the simpler ETM, both LM and BM provided good quality parameter estimates at SNR = 20, see for example the K^trans^ parameter in Fig 1A and 1B as compared to the exact values in Fig 1C. The parameter estimates in the ETM model are further improved with increasing SNR, resulting in smoother appearing images (results not shown). This observation is quantified in Fig 2A, where absolute bias and SD are presented for all ETM-based biomarkers, computed via the two algorithms as a function of SNR level. The curves are very similar for the two methods, showing very low bias (Fig 2A) and SD (Fig 2B) across SNR.

Fig 1J–1U display the face value of the parameters in the 2CXM at SNR = 20. Overall, the parameter estimates are observed to be close to the ground truth maps, see for instance the F_p_ parameter in Fig 1J–1L. Upon closer scrutiny, slight overestimation of F_p_ is observed for both LM and BM in the upper left corner, which corresponds to a situation with low plasma volume in conjunction with high flow. With a low v_p_, the amount of tracer passing through such a voxel is limited and the observed temporal signal intensity variation likewise. This, in turn, limits the contrast to noise ratio and ultimately results in bias towards overestimation of F_p_. The problem is alleviated to some extent with increasing SNR (results not shown). For the v_e_ maps (Fig 1M and 1N), one observes increasing bias from left to right compared to the ground truth. This area corresponds to decreasing intra- and extravascular flow in conjunction with constant accessible extravascular volume fraction. Hence, there is an inflow to the extravascular compartment determined by the AIF, but a slow washout, which makes it difficult to estimate parameters related to the tail of the concentration curve. This is more extensively explored in simulation studies in Appendix B in S1 File. Similar to the ETM, we present in Fig 2C and 2D mean relative bias and SD for the parameters in the two-compartment exchange model across SNR and observe, in general, quite fast convergence to low values for all parameters.

**Fig 3:**
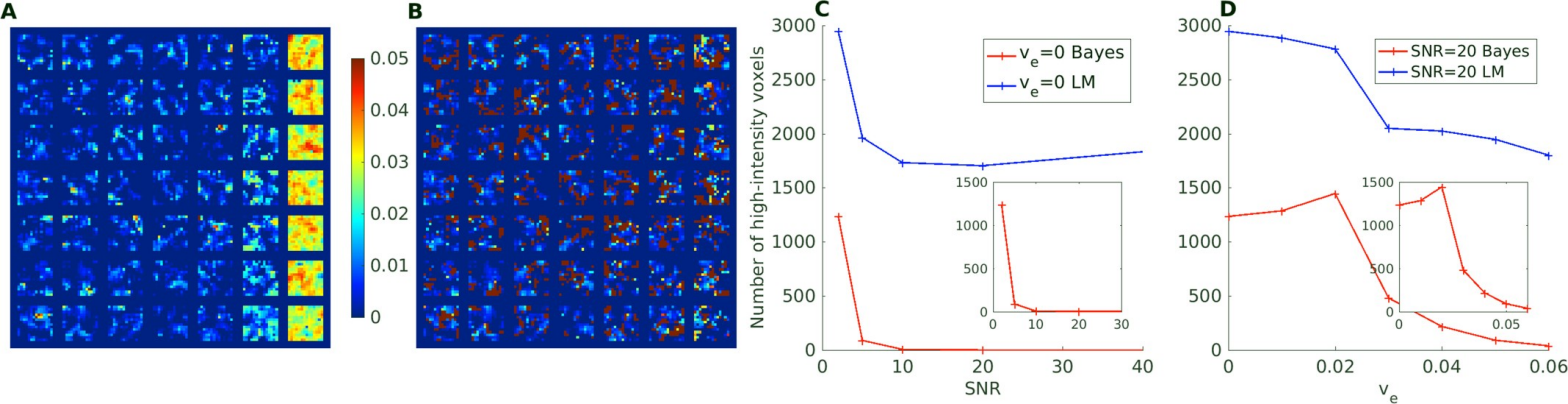
Illustration of v_e_ parameter estimation for very limited extravascular accessible volume. Panels A and B display the estimated 2XCM v_e_ parameter maps for a ground truth 2CXM model with v_e_ = 0. Panel C displays the number of severely overestimated voxels (defined as v_e_ greater than five times the ground truth value or 0.1 for v_e_ = 0) for the BM and LM methods at v_e_ = 0 as a function of SNR. Panel D displays the number of severely overestimated voxels as a function of ground truth v_e_ at SNR = 20.

One boundary condition, which is not directly probed in the simulations above, is the behavior in cases where the basic assumption of a two-compartment model is not met, i.e. where there is no possible transfer from the intra-vascular compartment to the extra-vascular compartment. This could be relevant in brain applications, where healthy tissue will have an intact brain-barrier, effectively nulling the extravasation. In Fig 3, we investigate situations with very limited to no extravasation in the 2CX model. Here, the LM based fitting method clearly overestimates the accessible extravascular volume fraction, especially for the special case with zero accessible volume, which might be an artifact of the parameters adjusting unfavorably to the curve. The extent of these spurious high-intensity voxels is reduced for increasing extravasation and increasing SNR, as illustrated by the plot in panels C and D of Fig 3. The BM methodology is observed to capture these situations better, with almost no voxels having v_e_ values above five times the ground truth value (0.1 is used for the v_e_ = 0 case) even for low SNRs.

### In vivo DCE data

**Fig 4:**
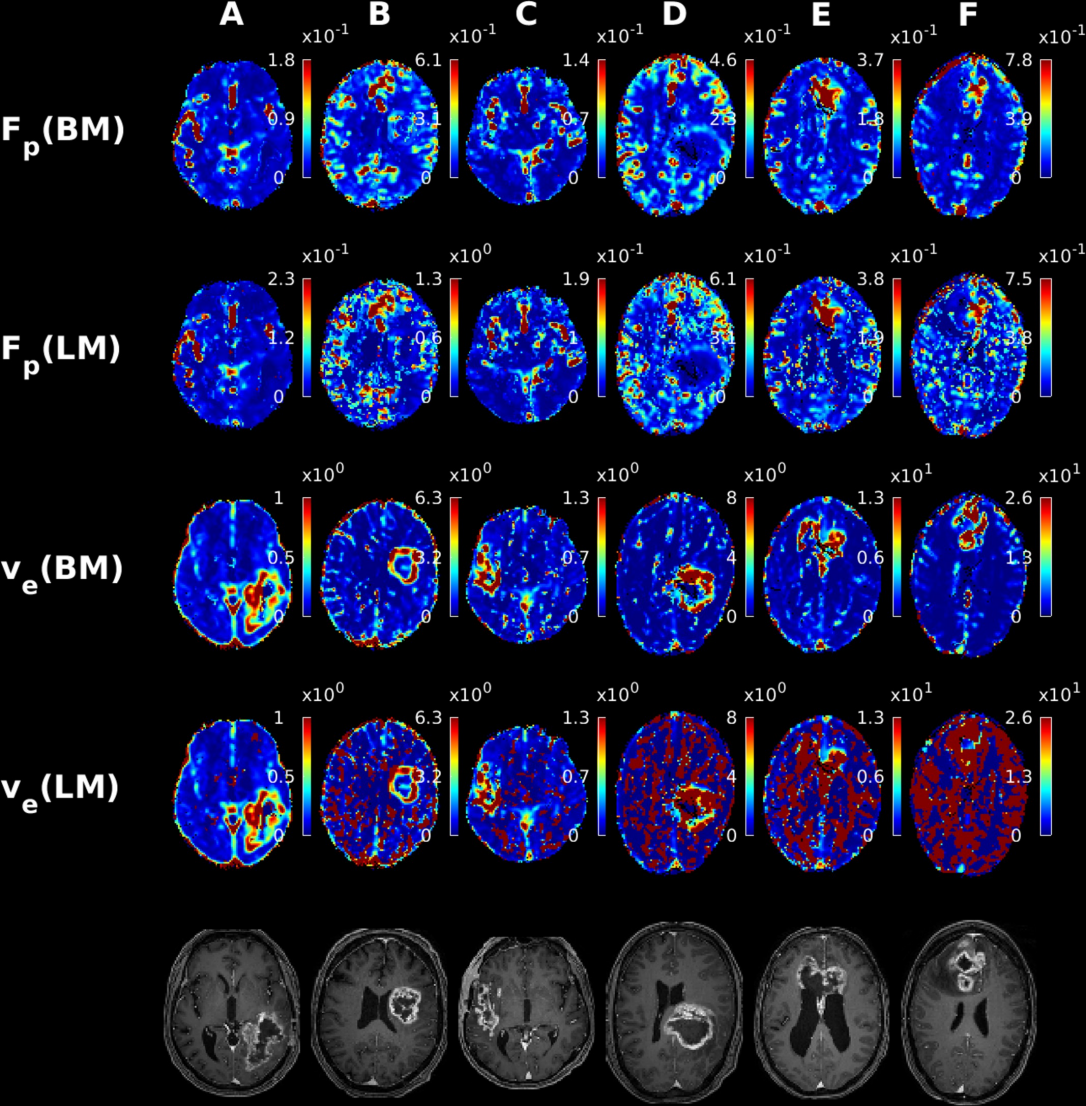
Examples of F_p_ and ve parameter maps from the 2CXM for six patients diagnosed with glioblastoma multiforme computed using the Bayes and LM algorithms. For reference the post contrast T1 weighted images registered to DCE space are displayed in the bottom row.

Fig 4 shows post-contrast T1-weighted images, 2CXM-based F_p_ and v_e_ parameter maps of six patients with GBMs, calculated using either LM or BM. The F_p_ maps are of similar quality for both algorithms, although the LM-based maps for subjects B, D, E, and F appear more scattered than their BM-based counterparts. For the v_e_ maps, however, the BM-based images stand out by their clarity with the tumor easily detectable against surrounding tissue in all cases. The LM-based v_e_ maps, on the other hand, vary substantially in quality, deteriorating notably from patient A to F in Fig 4. The tumor is adequately visible on the LM-based v_e_ maps in case A, apart from some scattered high intensity voxels, which might be due to limited signal intensity change in those areas. In cases B and C, the tumor is still discernible, although the high intensity voxels are much more prevalent. In patients D-F a vast number of high-intensity voxels lead to severe image degradation that prevents overall tumor outlining. In comparison, the tumor is clearly discernible on the corresponding BM-based v_e_ maps in all cases. Indeed, paired t-tests show significantly fewer extreme-intensity voxels for BM compared to LM for both ETM (p<0.0001) and 2CXM (p<0.0001). This superiority of BM may be attributed to the greater robustness of the algorithm towards voxels with very limited extravascular accessible volume. We interpret the v_e_ maps in D-F as measured examples of the corresponding phenomenon observed in the digital phantom simulations (see Fig 3), where severe overestimation of ve was more often encountered for the LM method compared to the BM method.

**Fig 5:**
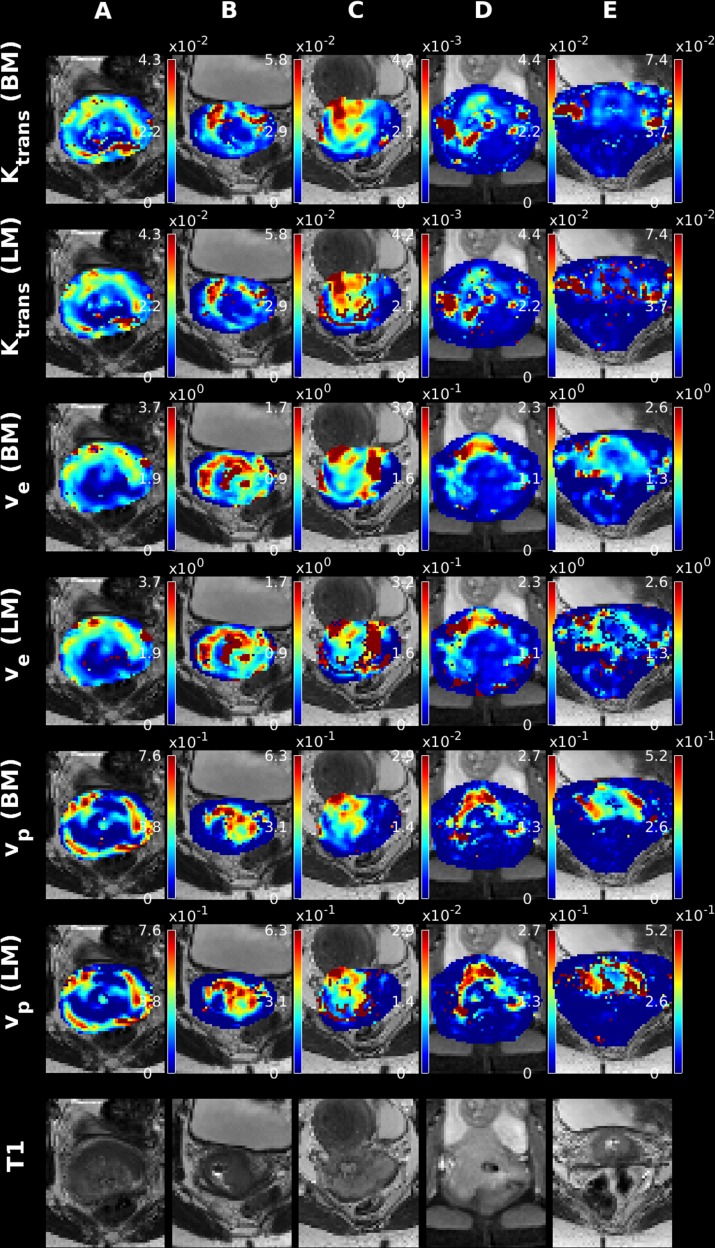
Examples of ETM parameter maps for 5 subjects diagnosed with cervical cancer, using the LM and Bayes algorithms.

**Fig 6:**
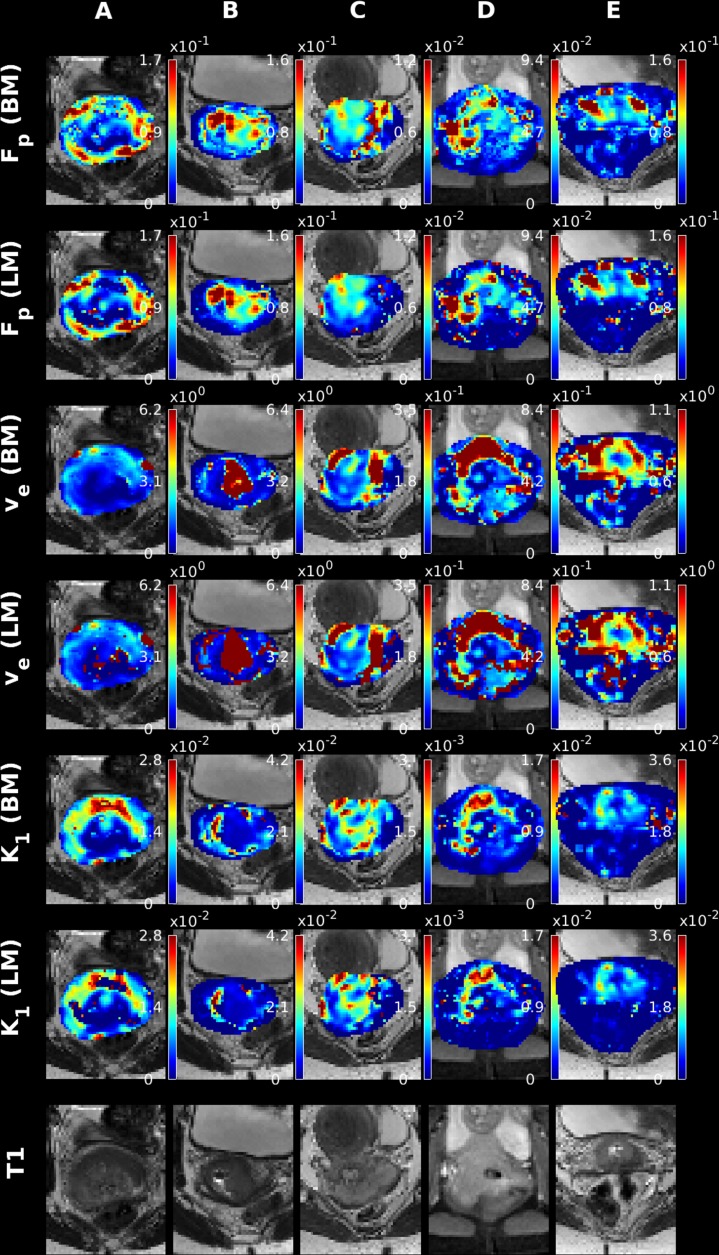
Examples of 2CXM parameters maps for 5 subjects diagnosed with cervical cancer using the LM and Bayes algorithms.

Since DCE imaging can be utilized outside of the brain, it is a natural next step to apply the presented algorithm to data obtained for non-brain tissue. Accordingly, we present in Fig 5 and Fig 6 five examples of parametric maps obtained with the LM and Bayesian methods. Fig 5 and Fig 6 display the results obtained for the ETM and 2CXM models, respectively. From Fig 5 and Fig 6, one initially observes quite similar appearing maps regardless of fitting algorithm used. Especially the high intensity scatter observed for the LM v_e_ parameter in the 2CXM is virtually non-existent in the cervical maps. This might be attributed to the limited view, which essentially covers only the tumor, and hence a reasonable SNR level is attained. Another possibility is the very different nature of brain tissue and non-brain tissue, where extreme extra-vascular transit times are not encountered, due to faster washout of extra-vascular tracer.

## Discussion

In this work, two curve-fitting algorithms for fitting well-established pharmacokinetic models to DCE MRI data were compared. We assessed a proposed Bayesian method, which has previously been shown to improve the diagnostic quality of DSC parameter maps [17], and compared the results to the standard LM approach, which is implemented in most conventional DCE MRI analysis tools.

For the ETM, we found that the parameter maps computed on simulated data were very similar regardless of fitting algorithm. We speculate that the ability for standard algorithms to provide good parameter estimates regardless of noise might provide an explanation for the extensive use of this method in the DCE analysis products marketed today, despite the suggested superiority of more complex models.

In cases characterized by a combination of high intra-/extravascular flow and a large extravascular volume, both algorithms performed poorly when estimating 2CXM-based parameters related to the extravascular compartment. The BM and LM methods provided quite different results in this case, where the BM consistently underestimated v_e_, while LM yielded more binary results. We speculate that the more stable performance, although underestimating the v_e_ parameter, might be caused by either the implicit parameter regularization through the prior covariance matrix or by the fact that the algorithms optimize substantially different functions. While the LM method minimizes a sum-of-squares residual, BM maximizes the posterior (log-)likelihood of the parameters given the observed data. It is possible that the (log-)likelihood transformation of the problem might result in a more stable multi-dimensional landscape for parameter estimation. The possible clinical consequences of the very slow extravascular dynamics are difficult to assess. It is conceivable that these extreme cases might not be encountered in practice but are simply a consequence of non-physiological combinations of model parameters. To that end, the simulation in the work of Sourbron *et al* [31] was limited to extravascular transit-times of at most 750s, which is well below the range used in our simulations (extravascular transit-times up to 3500s). However, it does suggest that care should be taken when selecting the acquisition duration in DCE experiments, since a too short scan time might severely impair the reliability of extra-vascular parameter maps. Parameters such as blood flow, blood volume, and plasma volume, are negligibly affected by the slow extravascular dynamics, in simulations and patient data alike. This can be ascribed to the fact that these parameters are primarily an effect of the initial part of the bolus passage, while the tail of the concentration curve largely determines the extra-vascular parameters.

Further, we presented parameter maps of six patients with high-grade gliomas, where DCE imaging has previously been proposed to be a source of clinical value [32–34], as well as five patients diagnosed with advanced stage cervical cancer. Quantitative DCE MRI is increasingly used for the assessment of for example pharmacodynamics changes in clinical trials [34]. The reliability of this technique has, however, been repeatedly questioned [35, 36], and we speculate that miscalculated voxels might considerably affect accurate parameter quantification. From the simulation results, we attribute this mainly to the differences between the two implementations in the event of very limited extravascular accessible volume.

The limitations of our study are primarily the retrospective design and the use of small sample sizes in the clinical data presented. However, the main goal of the present paper was first and foremost to characterize the performance of the Bayesian fitting algorithm and illustrate the value added compared to standard algorithms. We therefore refer to a clinical study published recently [37], investigating the clinical perception and diagnostic value of the generated parametric maps. In that work, we use a larger patient cohort and a systematic approach to evaluate the appearance of the parameter maps resulting from the two curve-fitting algorithms. Lastly, we note that improvement in image quality was observed in both brain and pelvic data, which gives some comfort in the generality of the method.

The algorithmic framework described in this work is general, albeit the prior distribution of the covariance matrix must be specified. In this, and related work, we specify the prior covariance as a diagonal matrix with the elements specified from empirically observed parameter variations. The algorithm is, however, rather robust against the exact specification of the prior covariance matrix [17], since the target posterior distribution is anyway estimated from data, and does not, in our experience, pose a limitation in practical application.

In this work, we have chosen to fit the pharmacokinetic models to the concentration curves rather than the signal intensity curves. Clearly this introduces a non-linear conversion of the image noise, making the noise distribution of the resulting CTCs extremely complicated and its derivation beyond the scope of this work. To make an operational algorithm, we have chosen to model the residual noise as drawn from a normal distribution, which is likely too simplistic. Conversely, the fitting to the observed signal change would require a further non-linear transformation of the parameters. This strategy provides an even more complex model to estimate and might be more error prone, since one part of it would contain the exponential to the convolution described in Eqs (1)–(3).

We note that we have limited the discussion to the so-called fast exchange limit (FXL) case, where water exchange between different compartments within an imaged voxel is assumed sufficiently fast. This is clearly not the case in actual tissue, where almost any voxel is compartmentalized. However, selection of pharmacokinetic model and exchange regime provide a plethora of different possibilities and indeed selecting the most appropriate model, albeit scientifically interesting [10–12], is a voxel-wise problem, which is incompatible with clinical routine. In this work, we have aimed at providing a reliable parameter estimation method, which should be sufficiently robust to be clinically applicable. This does not preclude the use of the algorithmic framework on more complex problems, but this is beyond the scope of this paper.

In conclusion, we presented and evaluated the performance of a new curve-fitting algorithm for estimating perfusion biomarkers in DCE MRI, which, in the case of the two-compartment exchange model, was found to be superior to the standard LM fitting algorithm typically employed for analysis of such data. We showed that the new BM approach improves the reliability of particularly v_e_ parameter maps, which was illustrated through application to data from brain tumor and cervical cancer patients. We found that the Bayesian method allowed the use of more elaborate models compared to the standard LM approach, which might ultimately open for clinical use of these models.

In addition, our findings illustrate that digital phantom simulations provide a solid and reliable method for evaluating the performance of a proposed algorithm. In addition, insights into the limitations and underlying mechanisms of a method may be gained. For example, it was found that long extravascular transit time might be prohibitive for obtaining reliable 2CXM parameters.

## Supporting information

S1 File Robust estimation of hemo-dynamic parameters in traditional DCE-MRI models. (PDF)

## References

[1] Campbell BCV, Mitchell PJ, Kleinig TJ, Dewey HM, Churilov L, Yassi N, et al Endovascular Therapy for Ischemic Stroke with Perfusion-Imaging Selection. New Engl J Med. 2015;372(11):1009–18. 10.1056/NEJMoa1414792 25671797

[2] Shiroishi MS, Habibi M, Rajderkar D, Yurko C, Go JL, Lerner A, et al Perfusion and permeability MR imaging of gliomas. Technol Cancer Res Treat. 2011;10:59–71. 10.7785/tcrt.2012.500180 21214289

[3] Donahue KM, Burstein D, Manning WJ, Gray ML. Studies of Gd-DTPA relaxivity and proton exchange rates in tissue. Magnetic Resonance in Medicine. 1994;32(1):66–76. 8084239 10.1002/mrm.1910320110

[4] Sommer JC, Schmid VJ. Spatial two-tissue compartment model for dynamic contrast-enhanced magnetic resonance imaging. J R Stat Soc C-Appl. 2014;63(5):695–713.

[5] Tofts PS. Modeling tracer kinetics in dynamic Gd-DTPA MR imaging. Jmri-J Magn Reson Im. 1997;7(1):91–101. 10.1002/jmri.1880070113 9039598

[6] Sourbron SP, Buckley DL. Tracer kinetic modelling in MRI: estimating perfusion and capillary permeability. Phys Med Biol. 2012;57(2):R1–R33. 10.1088/0031-9155/57/2/R1 22173205

[7] Sourbron S, Ingrisch M, Siefert A, Reiser M, Herrmann K. Quantification of Cerebral Blood Flow, Cerebral Blood Volume, and Blood-Brain-Barrier Leakage with DCE-MRI. Magnetic Resonance in Medicine. 2009;62(1):205–17. 10.1002/mrm.22005 19449435

[8] Patlak CS, Blasberg RG, Fenstermacher JD. Graphical Evaluation of Blood-to-Brain Transfer Constants from Multiple-Time Uptake Data. J Cereb Blood Flow Metab. 1983;3(1):1–7. 10.1038/jcbfm.1983.1 6822610

[9] Tofts PS, Kermode AG. Measurement of the Blood-Brain-Barrier Permeability and Leakage Space Using Dynamic Mr Imaging. 1. Fundamental-Concepts. Magnet Reson Med. 1991;17(2):357–67. 10.1002/mrm.1910170208 2062210

[10] Sommer JC, Gertheiss J, Schmid VJ. Spatially regularized estimation for the analysis of dynamic contrast-enhanced magnetic resonance imaging data. Stat Med. 2014;33(6):1029–41. 10.1002/sim.5997 24123120

[11] Duan C, Kallehauge JF, Bretthorst GL, Tanderup K, Ackerman JJH, Garbow JR. Are complex DCE-MRI models supported by clinical data? Magnetic Resonance in Medicine. 2016:n/a-n/a. 10.1002/mrm.26189 PMC5548456 26946317

[12] Li X, Welch EB, Chakravarthy AB, Xu L, Arlinghaus LR, Farley J, et al Statistical Comparison of DCE-MRI Pharmacokinetic Models in Human Breast Cancer. Magnetic Resonance in Medicine. 2012;68(1):261–71. 10.1002/mrm.23205 22127821 PMC3291742

[13] Dikaios N, Atkinson D, Tudisca C, Purpura P, Forster M, Ahmed H, et al A comparison of Bayesian and non-linear regression methods for robust estimation of pharmacokinetics in DCE-MRI and how it affects cancer diagnosis. Comput Med Imag Grap. 2017;56:1–10. 10.1016/j.compmedimag.2017.01.003 28192761

[14] Yang C, Karczmar GS, Medved M, Oto A, Zamora M, Stadler WM. Reproducibility Assessment of a Multiple Reference Tissue Method for Quantitative Dynamic Contrast Enhanced-MRI Analysis. Magnet Reson Med. 2009;61(4):851–9. 10.1002/mrm.21912 PMC2758034 19185002

[15] Heye T, Merkle EM, Reiner CS, Davenport MS, Horvath JJ, Feuerlein S, et al Reproducibility of Dynamic Contrast-enhanced MR Imaging Part II. Comparison of Intra- and Interobserver Variability with Manual Region of Interest Placement versus Semiautomatic Lesion Segmentation and Histogram Analysis. Radiology. 2013;266(3):812–21. 10.1148/radiol.12120255 23220891

[16] Heye T, Davenport MS, Horvath JJ, Feuerlein S, Breault SR, Bashir MR, et al Reproducibility of Dynamic Contrast-enhanced MR Imaging Part I. Perfusion Characteristics in the Female Pelvis by Using Multiple Computer-aided Diagnosis Perfusion Analysis Solutions. Radiology. 2013;266(3):801–11. 10.1148/radiol.12120278 23220897

[17] Mouridsen K, Hansen MB, Ostergaard L, Jespersen SN. Reliable estimation of capillary transit time distributions using DSC-MRI. J Cereb Blood Flow Metab. 2014;34(9):1511–21. 10.1038/jcbfm.2014.111 24938401 PMC4158667

[18] Kallehauge J, Nielsen T, Haack S, Peters DA, Mohamed S, Fokdal L, et al Voxelwise comparison of perfusion parameters estimated using dynamic contrast enhanced (DCE) computed tomography and DCE-magnetic resonance imaging in locally advanced cervical cancer. Acta Oncol. 2013;52(7):1360–8. 10.3109/0284186X.2013.813637 24003852

[19] Hansen MB, Tietze A, Kalpathy-Cramer J, Gerstner ER, Batchelor TT, Østergaard L, et al Reliable Estimation of Microvascular Flow Patterns in Patients with Disrupted Blood-Brain-Barrier using DSC-MRI. J Magn Reson Imag. 2016. 10.1002/jmri.25549 27902858

[20] Schmid VJ, Whitcher B, Padhani AR, Taylor NJ, Yang GZ. Bayesian methods for pharmacokinetic models in dynamic contrast-enhanced magnetic resonance imaging. Ieee T Med Imaging. 2006;25(12):1627–36. 10.1109/tmi.2006.884210 17167997

[21] Schmid VJ, Whitcher B, Yang GZ. Semi-parametric analysis of dynamic contrast-enhanced MRI using Bayesian P-splines. Lect Notes Comput Sc. 2006;4190:679–86. 10.1007/11866565_83 17354949

[22] Parker GJM, Roberts C, Macdonald A, Buonaccorsi GA, Cheung S, Buckley DL, et al Experimentally-derived functional form for a population-averaged high-temporal-resolution arterial input function for dynamic contrast-enhanced MRI. Magnet Reson Med. 2006;56(5):993–1000. 10.1002/mrm.21066 17036301

[23] Dikaios N, Arridge S, Hamy V, Punwani S, Atkinson D. Direct parametric reconstruction from undersampled (k, t)-space data in dynamic contrast enhanced MRI. Med Image Anal. 2014;18(7):989–1001. 10.1016/j.media.2014.05.001 24972377

[24] Orton MR, Collins DJ, Walker-Samuel S, d'Arcy JA, Hawkes DJ, Atkinson D, et al Bayesian estimation of pharmacokinetic parameters for DCE-MRI with a robust treatment of enhancement onset time. Phys Med Biol. 2007;52(9):2393–408. 10.1088/0031-9155/52/9/005 17440242

[25] Sharma P, Socolow J, Patel S, Pettigrew RI, Oshinski JN. Effect of Gd-DTPA-BMA on blood and myocardial T1 at 1.5T and 3T in humans. Journal of Magnetic Resonance Imaging. 2006;23(3):323–30. 10.1002/jmri.20504 16456820 PMC7166832

[26] Lu H, Clingman C, Golay X, van Zijl PCM. Determining the longitudinal relaxation time (T1) of blood at 3.0 Tesla. Magnetic Resonance in Medicine. 2004;52(3):679–82. 10.1002/mrm.20178 15334591

[27] Stanisz GJ, Odrobina EE, Pun J, Escaravage M, Graham SJ, Bronskill MJ, et al T-1, T-2 relaxation and magnetization transfer in tissue at 3T. Magnetic Resonance in Medicine. 2005;54(3):507–12. 10.1002/mrm.20605 16086319

[28] Gudbjartsson H, Patz S. The Rician Distribution of Noisy MRI Data. Magnetic resonance in medicine: official journal of the Society of Magnetic Resonance in Medicine / Society of Magnetic Resonance in Medicine. 1995;34(6):910–4. 10.1002/mrm.1910340618 PMC2254141 8598820

[29] Louis DN, Ohgaki H, Wiestler OD, Cavenee WK, Burger PC, Jouvet A, et al The 2007 WHO Classification of Tumours of the Central Nervous System. Acta Neuropathologica. 2007;114(2):97–109. 10.1007/s00401-007-0243-4 17618441 PMC1929165

[30] Haase A, Frahm J, Matthaei D, Hanicke W, Merboldt KD. FLASH imaging. Rapid NMR imaging using low flip-angle pulses. Journal of Magnetic Resonance (1969). 1986;67(2):258–66. 10.1016/j.jmr.2011.09.021 22152368

[31] Luypaert R, Sourbron S, Makkat S, de Mey J. Error estimation for perfusion parameters obtained using the two-compartment exchange model in dynamic contrast-enhanced MRI: a simulation study. Phys Med Biol. 2010;55(21):6431–43. 10.1088/0031-9155/55/21/006 20952813

[32] Bisdas S, Naegele T, Ritz R, Dimostheni A, Pfannenberg C, Reimold M, et al Distinguishing recurrent high-grade gliomas from radiation injury: a pilot study using dynamic contrast-enhanced MR imaging. Acad Radiol. 2011;18:575–83. 10.1016/j.acra.2011.01.018 21419671

[33] Patankar TF, Haroon HA, Mills SJ, Baleriaux D, Buckley DL, Parker GJM, et al Is volume transfer coefficient (K(trans)) related to histologic grade in human gliomas? Am J Neuroradiol. 2005;26(0195–6108 (Print)):2455–65. 16286385 PMC7976173

[34] Sorensen AG, Batchelor TT, Zhang W-T, Chen P-J, Yeo P, Wang M, et al A "vascular normalization index" as potential mechanistic biomarker to predict survival after a single dose of cediranib in recurrent glioblastoma patients. Cancer Res. 2009;69:5296–300. 10.1158/0008-5472.CAN-09-0814 19549889 PMC2824172

[35] Huang W, Li X, Chen Y, Li X, Chang M-C, Oborski MJ, et al Variations of Dynamic Contrast-Enhanced Magnetic Resonance Imaging in Evaluation of Breast Cancer Therapy Response: A Multicenter Data Analysis Challenge. Translational Oncology. 2014;7(1):153–66. 24772219 10.1593/tlo.13838 PMC3998693

[36] Heye T, Davenport MS, Horvath JJ, Feuerlein S, Breault SR, Bashir MR, et al Reproducibility of dynamic contrast-enhanced MR imaging. Part I. Perfusion characteristics in the female pelvis by using multiple computer-aided diagnosis perfusion analysis solutions. Radiology. 2013;266(1527–1315 (Electronic)):801–11. 10.1148/radiol.12120278 23220897

[37] Tietze A, Nielsen A, Mikkelsen IK, Hansen MB, Obel A, Ostergaard L, et al Bayesian modeling of Dynamic Contrast Enhanced MRI data in cerebral glioma patients improves the diagnostic quality of hemodynamic parameter maps. Plos One. 2018;13(9). 10.1371/journal.pone.0202906 PMC6157834 30256797

