# Supplementary material for: Robust estimation of hemo-dynamic parameters in traditional DCE-MRI models
Source: PLoS One. 2019 Jan 3;14(1):e0209891. doi: 10.1371/journal.pone.0209891 (PMC6317807; doi:10.1371/journal.pone.0209891)
Supplement: S1 File — (PDF) [file pone.0209891.s001.pdf]

Supporting Information S1: Robust estimation of hemodynamic parameters in traditional DCE-MRI models

Mikkel B. Hansen, Anna Tietze, Søren Haack, Jesper Kallehauge, Irene K. Mikkelsen, Leif Østergaard, Kim Mouridsen

Appendix A: Conversion of signal to concentration

The observed dynamic MRI signal for a particular voxel can be converted into indicator concentration based on the MRI sequence used. In this work, we use two different sequences, which results in different equations relating the observed MRI signal to indicator concentration, (i) a steady-state sequence, and (ii) a centric saturation recovery sequence [1]. The signal-concentration formula for the steady-state sequence is given as

$$\text{Steady-state:} \quad [Gd] = -\frac{1}{T_R \times r_1} \log\left(\frac{\eta - 1}{\eta \cos\alpha - 1}\right)$$

where T_R it the repetition time, r_1 the so-called T1 relaxivity, α the flip angle, and η is an auxiliary variable,

$$\eta = \frac{\frac{S(t)}{S(0)}\left(1 - \exp\left(-T_R R_{1,0}\right)\right)}{1 - \exp\left(-T_R R_{1,0}\right)\cos\alpha}$$

where S(0) is the observed signal before contrast injection, S(t) the observed signal at time t, and $R_{1,0}$ the pre-contrast relaxation rate. For the saturation recovery sequence used in the present study, the tracer concentration may be obtained from the saturation recovery signal equation (see e.g. [2]), and is given as [1]

$$\text{Saturation recovery:} \quad [Gd] = \frac{-\log\left(\left(1-\exp\left(-T_{sat}R_{1,0}\right)\right)\times\left(\frac{S(t)}{S(0)}+\frac{\exp\left(-T_{sat}R_{1,0}\right)}{1-\exp\left(-T_{sat}R_{1,0}\right)}\right)\right)}{r_1 \times T_{sat}}$$

where T_{sat} is the so-called saturation time, while the remaining elements are defined above.

Appendix B: Effect of acquisition duration

We now provide a more detailed analysis of the v_e underestimation observed for the 2CXM, in terms of computed extra-vascular transit times T_E. In

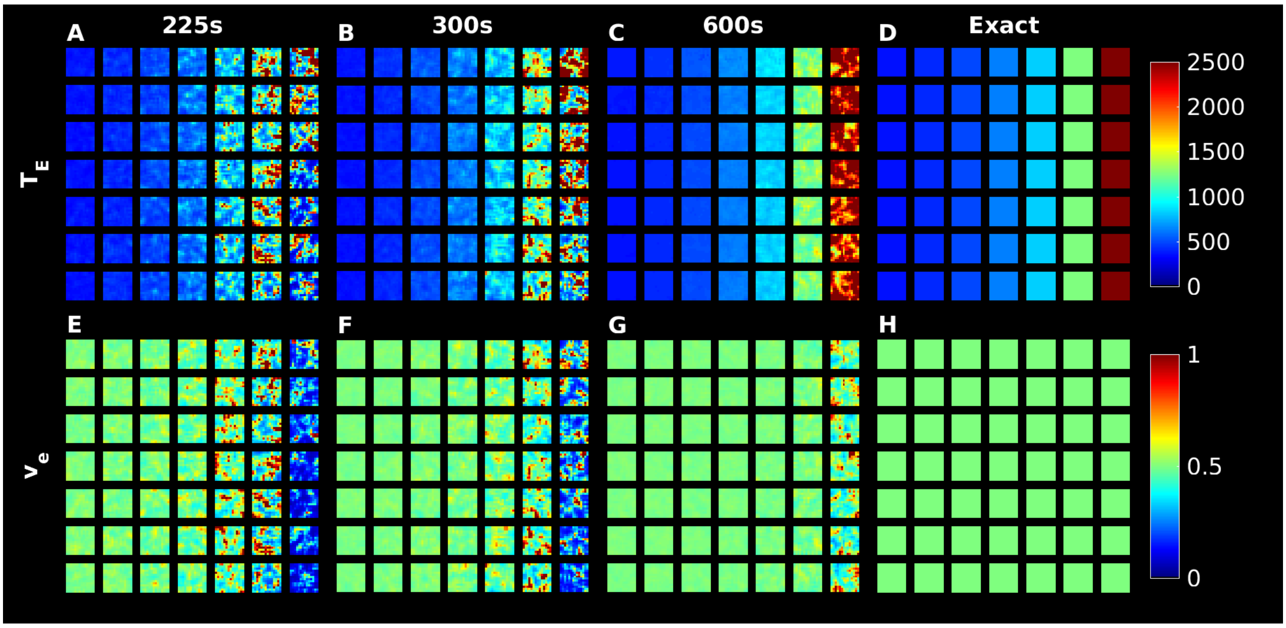

Figure 1 A-C, we present T_E maps (SNR=40), as they appear when calculated with the Bayesian algorithm, where the difference between maps is the acquisition duration. These can be compared to the exact reference, which is presented in

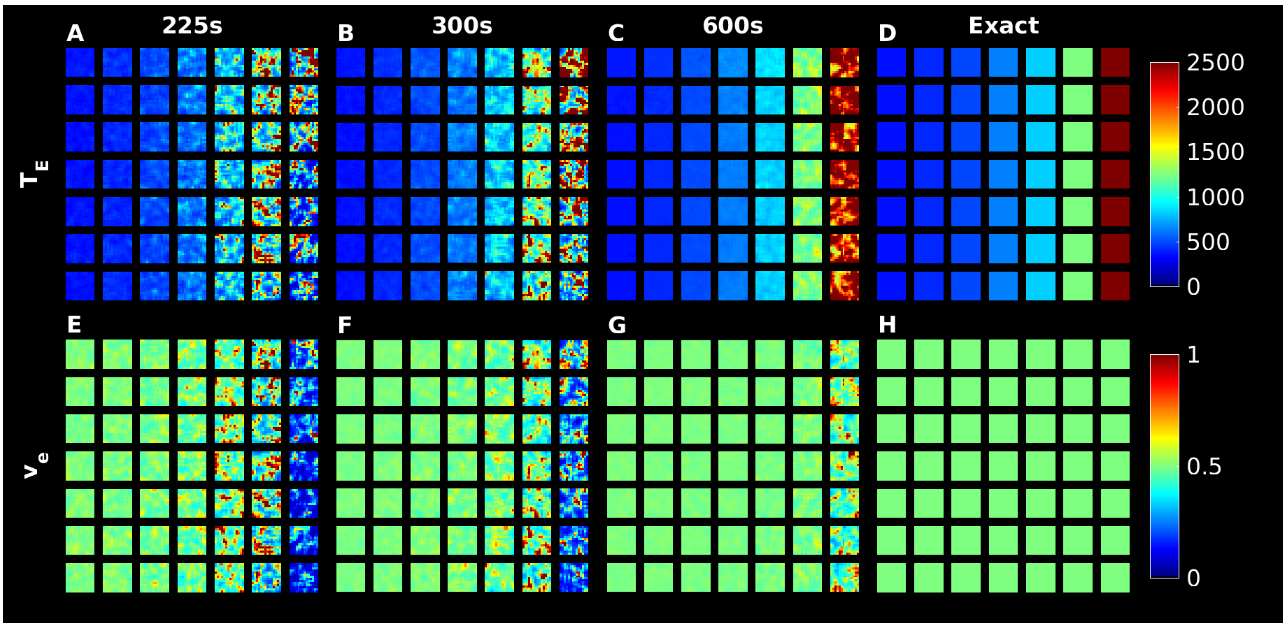

Figure 1 D. The corresponding v_e maps are presented in

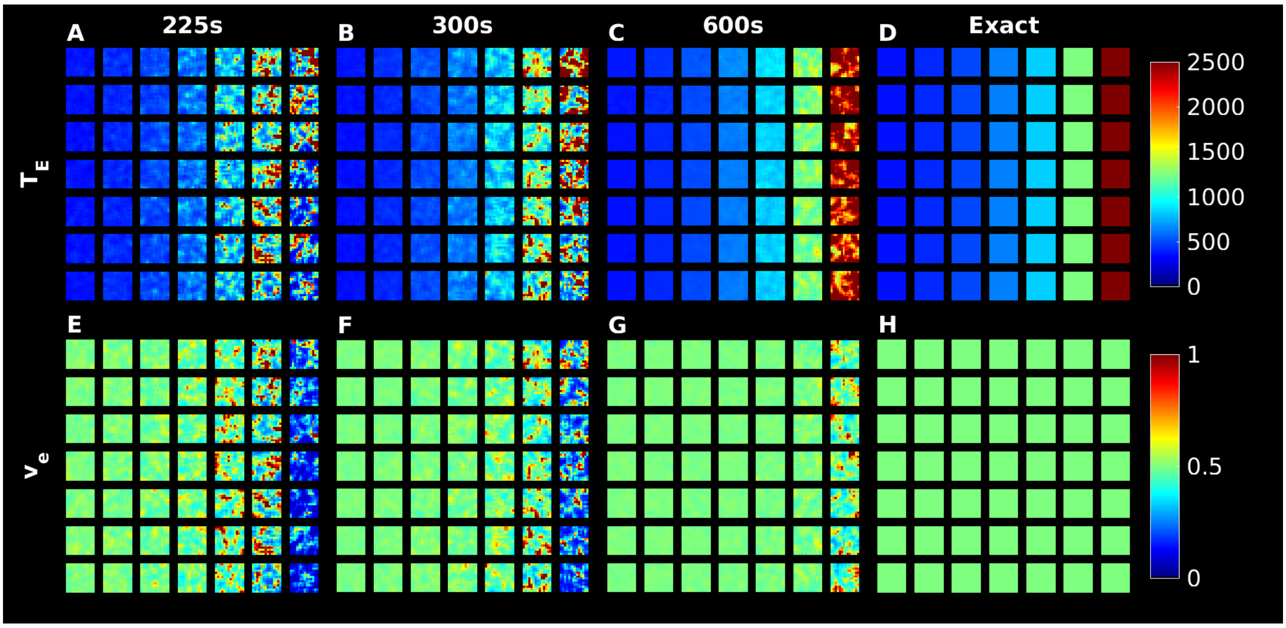

Figure 1 E-H. Clearly, the quality of both T_E and v_e maps is markedly improved in the right-hand side of the phantoms when increasing the acquisition duration. This area corresponds to voxels with very high extra-vascular transit-times, which in turn means that the indicator washout from the extra-vascular space is extremely slow. Simultaneously, the inflow is non-zero over a long period of time owing to the slow dilution of intravascular tracer (non-zero tail of the AIF). The combination

of these phenomena results in an appearing monotonically increasing tracer concentration in the extravascular compartment, when measured with short acquisition durations. This is illustrated in Figure 2, where the tracer concentration curve is observed to be increasing throughout the scan duration (red curve). In the short acquisition case (

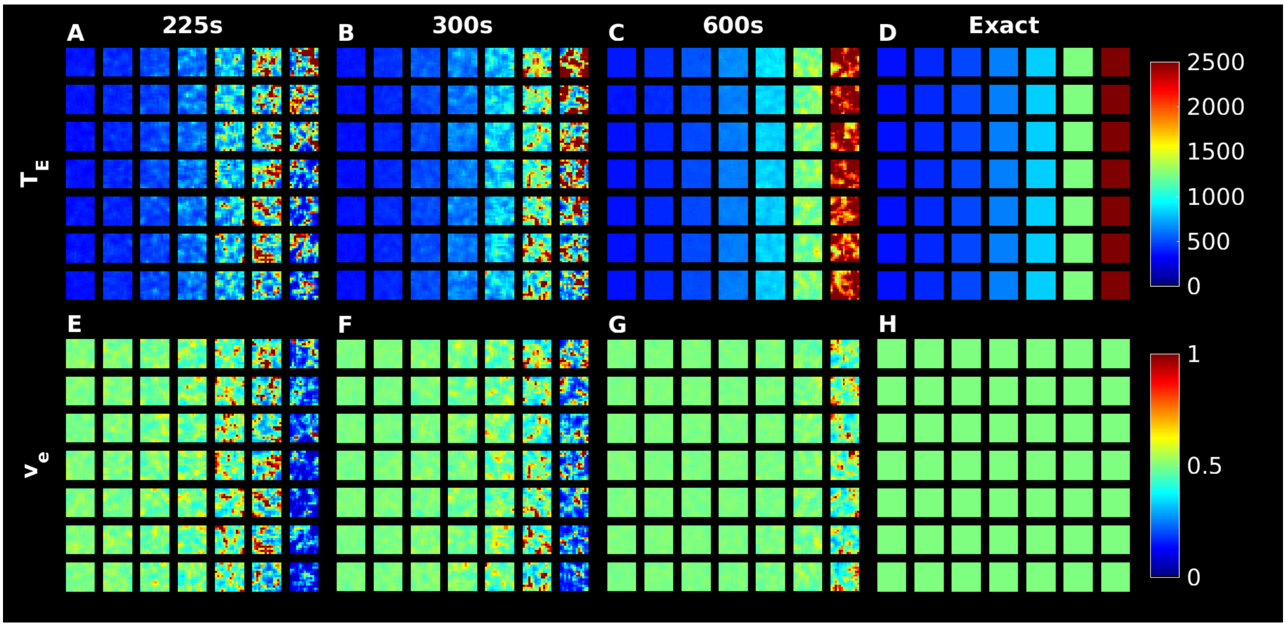

Figure 1 A and E) only the initial 3-4 minutes of the bolus passage is covered, which is insufficient for obtaining an accurate representation of areas with such slow extra-vascular dynamics. Overall, the maps are quite similar, but the LM results in the rightmost column appear more binary compared to the BM counterpart. We attribute this observation to the combination of slow extravascular dynamics, as described above, and the specific appearance of the tail in individual noise realizations. Removing this stochastic element, i.e. creating a noise-free phantom, makes the BM and LM maps virtually identical (results not shown), which suggests that BM is more robust against random noise.

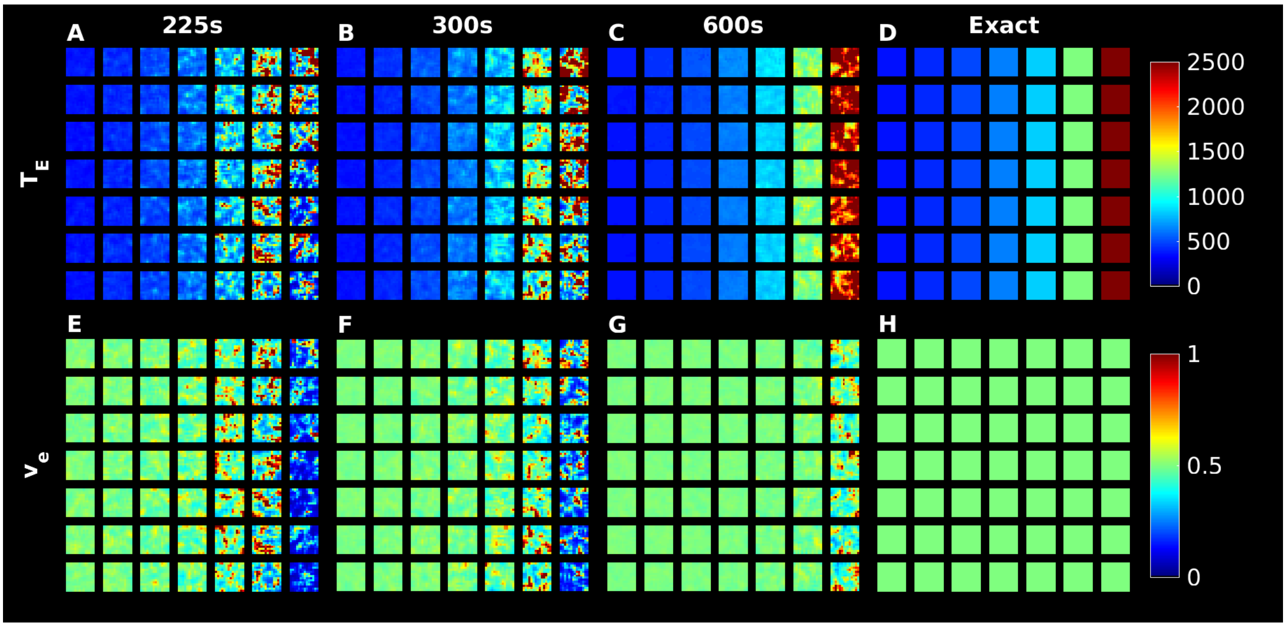

Figure 1: Illustration of the effect of scan duration on extra-vascular transit time (T_E) and v_e computation. Panels A-C display the T_E maps from increasingly longer scan durations, with the ground truth is displayed in panel D. The corresponding v_e maps are presented in panels E-H. All estimated results are based on the Bayes algorithm.

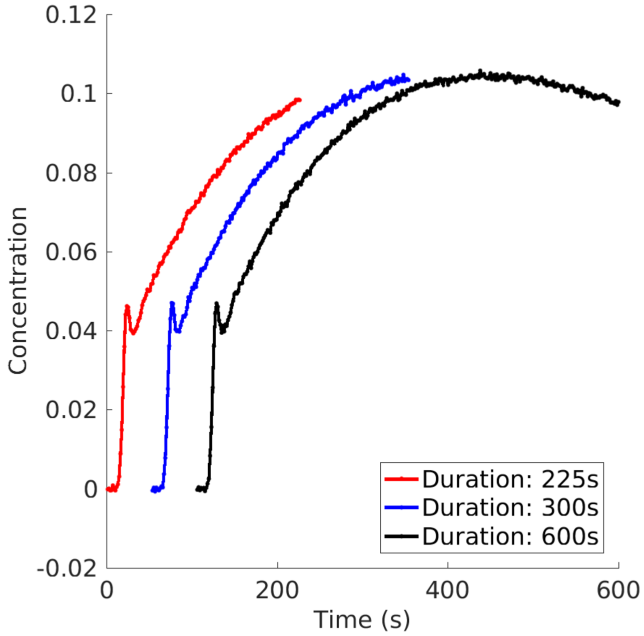

Figure 2: Illustration of tracer concentration as a function of time. The red, blue, and black curves correspond to simulated scan durations of 225, 300, and 600 seconds, respectively. Note that the blue and red curves have been shifted by 10 seconds to allow better individual visualization.

Appendix C: T1 measurement and effects on quantification of hemodynamic parameters in DCE

In Figure 3, we illustrate the consequences of using incorrect T1 baseline values (T1(0)) in the concentration curve generation, compared to the T1 values resulting in the observed tissue concentration curve. The arterial T1 value is in all cases 1.66s, in simulation and estimation alike. The tissue T1 is, however, fixed in the estimation procedure to 0.5s (second row in Figure 3, denoted 'Shorter T1'), 1s ('Correct T1'), and 3s ('Longer T1'), respectively, while the tissue curves are simulated using a T1 value of 1s. Figure 3 displays only maps generated with the Bayesian algorithm, but we note that the maps from the LM algorithm are of similar quality. Initially, one observes that the maps generated by assuming the correct T1 values (second row in Figure 3) are virtually identical to the ground truth (top row in Figure 3). However, using longer (third row) or shorter (fourth row)

T1 values in the estimation procedure dramatically changes the appearance of all parameter maps. Specifically, the use of a longer T1 value consistently underestimates parameters, while a shorter T1 value overestimates parameters.

We speculate that this observed drastic sensitivity of the appearance of the parameter maps for the steady-state experiment, when using incorrect T1 values, might represent a potential source of uncertainty in clinical application, since one might conceivably both over- and underestimate parameters, thus resulting in incorrectly appearing pathology. In the case of cerebral tumors, where steady-state DCE has been a suggested source of added clinical value [3, 4] the problem may, however, be alleviated to some extent, since tumoral T1 values are often longer than T1 values in normal appearing tissue. Hence, the parameter maps might appear qualitatively correct [5], despite the overestimation of parameters. Saturation-recovery data sampled and processed as in Kallehauge et al [1] are much less affected by incorrect T1 values.

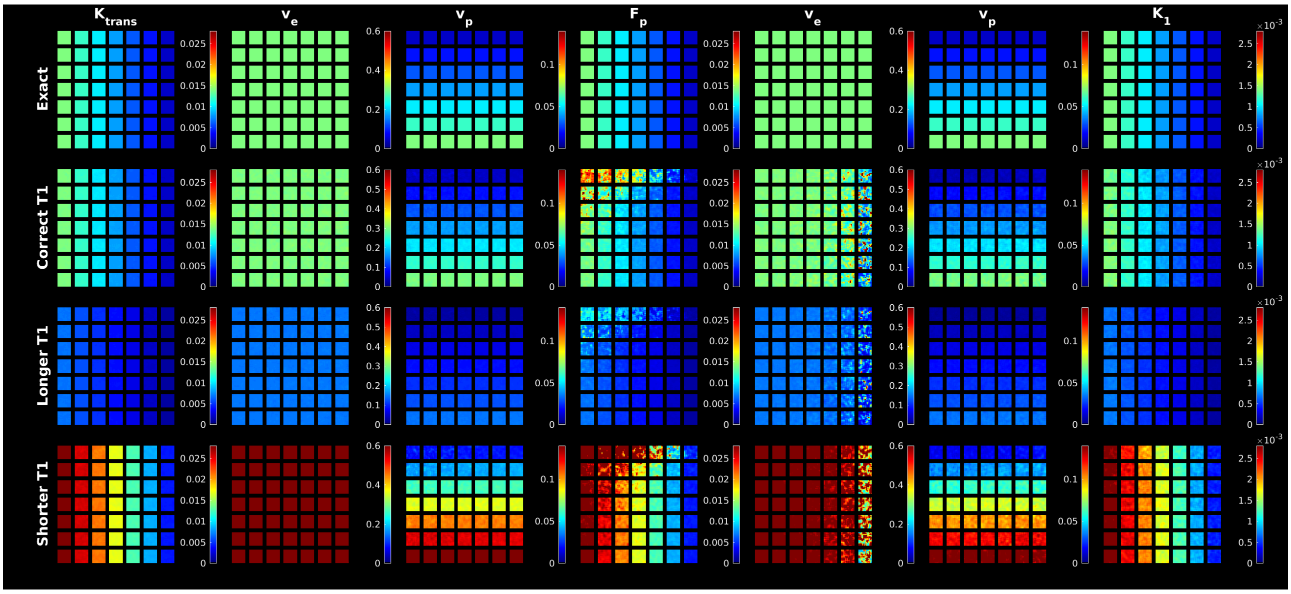

Figure 3: Simulations of the use of incorrect T1 values in the generation of concentration curves, illustrating the effect on the fitted parameter maps. The parameters K^{trans}, v_e, and v_p are from the

ETM, while F_p, v_e, v_p, and K_1 are from the 2CXM. All maps are calculated using the Bayes algorithm. The experiment simulated is a steady-state DCE measurement.

References

1. Kallehauge J, Nielsen T, Haack S, Peters DA, Mohamed S, Fokdal L, et al. Voxelwise comparison of perfusion parameters estimated using dynamic contrast enhanced (DCE) computed tomography and DCE-magnetic resonance imaging in locally advanced cervical cancer. Acta Oncologica. 2013;52(7):1360-8.
2. Larsson HB, Hansen Ae Fau - Berg HK, Berg Hk Fau - Rostrup E, Rostrup E Fau - Haraldseth O, Haraldseth O. Dynamic contrast-enhanced quantitative perfusion measurement of the brain using T1-weighted MRI at 3T. J Magn Reson Imaging. 2008;27(4):754-62.
3. Mills SJ, du Plessis D, Pal P, Thompson G, Buonacorrsi G, Soh C, et al. Mitotic Activity in Glioblastoma Correlates with Estimated Extravascular Extracellular Space Derived from Dynamic Contrast-Enhanced MR Imaging. American Journal of Neuroradiology. 2016;37(5):811-7.
4. Yun TJ, Park CK, Kim TM, Lee SH, Kim JH, Sohn CH, et al. Glioblastoma Treated with Concurrent Radiation Therapy and Temozolomide Chemotherapy: Differentiation of True Progression from Pseudoprogression with Quantitative Dynamic Contrast-enhanced MR Imaging. Radiology. 2015;274(3):830-40.
5. Tietze A, Mouridsen K, Mikkelsen IK. The impact of reliable prebolus T 1 measurements or a fixed T 1 value in the assessment of glioma patients with dynamic contrast enhancing MRI. Neuroradiology. 2015;57(6):561-72.
